# Immunomodulatory germline variation associated with the development of multiple primary melanoma (MPM)

**DOI:** 10.1038/s41598-019-46665-z

**Published:** 2019-07-15

**Authors:** Robert Ferguson, Alexi Archambault, Danny Simpson, Leah Morales, Vylyny Chat, Esther Kazlow, Rebecca Lax, Garrett Yoon, Una Moran, Richard Shapiro, Anna Pavlick, David Polsky, Iman Osman, Tomas Kirchhoff

**Affiliations:** 1 Perlmutter Cancer Center, New York University School of Medicine, New York, USA; 2 Departments of Population Health and Environmental Medicine, New York University School of Medicine, New York, USA; 3 The Interdisciplinary Melanoma Cooperative Group, New York University School of Medicine, New York, USA; 4 Department of Medicine, New York University School of Medicine, New York, USA; 5 Ronald O. Perelman, Department of Dermatology, New York University, New York, USA; 6 Department of Surgery, New York University School of Medicine, New York, USA; 7 Department of Pathology, New York University School of Medicine, New York, USA

**Keywords:** Melanoma, Cancer genetics

## Abstract

Multiple primary melanoma (MPM) has been associated with a higher 10-year mortality risk compared to patients with single primary melanoma (SPM). Given that 3–8% of patients with SPM develop additional primary melanomas, new markers predictive of MPM risk are needed. Based on the evidence that the immune system may regulate melanoma progression, we explored whether germline genetic variants controlling the expression of 41 immunomodulatory genes modulate the risk of MPM compared to patients with SPM or healthy controls. By genotyping these 41 variants in 977 melanoma patients, we found that rs2071304, linked to the expression of *SPI1*, was strongly associated with MPM risk reduction (OR = 0.60; 95% CI = 0.45–0.81; p = 0.0007) when compared to patients with SPM. Furthermore, we showed that rs6695772, a variant affecting expression of BATF3, is also associated with MPM-specific survival (HR = 3.42; 95% CI = 1.57–7.42; p = 0.0019). These findings provide evidence that the genetic variation in immunomodulatory pathways may contribute to the development of secondary primary melanomas and also associates with MPM survival. The study suggests that inherited host immunity may play an important role in MPM development.

## Introduction

Approximately 3–8% of patients diagnosed with cutaneous melanoma will develop additional primary melanomas during their lifetime^1,2^ (referred to as multiple primary melanoma, or MPM), which, may confer a higher 10-year mortality risk when compared to patients with a single primary melanoma (SPM)^3^. Despite an ongoing debate whether increased MPM incidence is due to improved surveillance, as we have also recently suggested^4^, the biological underpinning of MPM phenotype is poorly understood. While most patients with MPM present with two primary tumors^5^ (~80%), the diagnoses of more than two multiple melanomas are not uncommon^6^. To date, the known risk factors for MPM include a personal history of dysplastic nevi^7^ and a family history of melanoma, which only account for approximately 20% of all MPM diagnoses^5^, leaving vast majority of MPM etiology unexplained.

Several reports provided evidence that individuals with immunodeficiencies have a higher risk of cancer occurrence, including melanoma, as compared to the general population^8^. Studies on immunodeficient HIV patients with prior diagnoses of skin cancer have shown that these patients are at greater risk of skin cancer recurrence^9^. These observations, and the fact that melanomas are more immunogenic compared to other tumors, further suggest that the host immunity may impact melanomagenesis both in the context of metastatic progression as well as in its initial presentation and recurrence, hence increasing a risk for additional primary melanomas.

Recent genome-wide association studies (GWAS)^10–14^ and candidate pathway studies^15^ have identified germline genetic variants associated with cutaneous melanoma risk^10–13^; however, the literature on MPM-specific genetic risk markers remains scarce. While most melanoma GWAS analyses were performed on patient populations with predominantly single primary diagnoses, few GWAS studies^12,13^ investigated whether the presence of additional primaries modulated differential risk for specific variants. Besides GWAS, the candidate scans have been performed on known melanoma-related risk pathways and associations were found between polymorphisms in XPD^16^, TYR^17^, and MTAP^17^ and MPM risk. Other studies have examined associations between MPM risk and the pigmentation pathway by assessing variations in MC1R^18–20^ and MITF^21^, as well as vitamin D synthesis among individuals exposed to high levels of sunlight in recreational activities^22^, although these results were of borderline significance. However, besides these GWAS or candidate scans focused on genetic risk variation derived from predominantly SPM populations, the potentially novel MPM-specific risk loci have not been investigated. Also, to our knowledge, the genetic variation potentially impacting host immunity and its role in the development of additional primary tumors has not been explored. Recently, large datasets, such as the Multiple Tissue Human Expression Resource (MuTHER)^23^, linked the genetic variation with the gene expression in immune lymphoblastoid cells, allowing for opportunities to investigate the inherited basis of host immunity related to cancer outcomes. We have recently reported that the germline genetic variants linked with the expression of immunomodulatory genes (immunomodulatory expression quantitative trait loci, or ieQTLs) in lymphoblastoid cell lines (LCLs) are associated with melanoma recurrence^24^ and survival^25^. Here we hypothesize that MPM may be in part attributed to impaired immune regulation during melanomagenesis in patients with SPM, and this altered host immunity is determined by inherited genetic variation. In this study, using a cohort of 977 melanoma patients, including 147 MPM patients, we sought to assess the potential of 41 ieQTLs in LCLs from MuTHER as putative biomarkers of host immunity associated with the development of additional melanoma primaries. We have also tested whether ieQTLs differentially modulate survival in patients with MPM.

## Materials and Methods

### Study population

The study population included 977 patients of self-reported non-Hispanic European descent, with no reported family history of melanoma, treated between 2002 and 2013 at New York University Langone Health (NYULH) with stage I to III cutaneous melanoma, prospectively enrolled in the NYU Interdisciplinary Melanoma Cooperative Group’s Institutional (IMCG) Review Board-approved clinicopathological database and biorepository and actively followed up on a protocol-driven schedule^24,26,27^. Blood samples, demographic, follow-up, and clinical information including age at pathological diagnosis, gender, self-reported family history of melanoma, and 2009 AJCC staging at diagnosis, were collected as part of IMCG protocol, and all patients (parent or legal guardian for any patient under 18) provided written informed consent at time of enrollment. Subjects with a reported family history of melanoma were excluded from the final patient cohort. Control study participants consisted 1047 cancer-free non-Hispanic subjects from a cutaneous melanoma case-control study at MD Anderson Cancer Center from March 1998 to August 2008 (accession number phs000187.v1.p1) who were acquaintances of patients that presented at other clinics^14^. The methods in this study were carried out in accordance with guidelines set forth by the IRB at NYULH relating to the use of patient samples in genetic studies, and all experimental protocols were approved by NYULH.

### Selection of immunomodulatory eQTLs and genotyping

Previously, we utilized publicly available databases to identify 382 immunomodulatory genes and these loci were used to isolate candidate SNPs of interest for genotyping^25^. Employing resources from the MuTHER project^28,29^, 50 SNPs with the most significant cis-ieQTL activity on probes in LCLs from these immunomodulatory loci were selected for genotyping. While skin and adipose tissue data are also available in the MuTHER project, the cis-ieQTLs have been assessed from the LCL expression data, as the scope of the study is focused on the host immune cell component involved in MPM development. The selection procedure was described in detail elsewhere^25^.

Genomic DNA from all 977 cases was isolated from whole blood samples using a QiaAmp kit (Qiagen). All SNP genotyping was completed using the MassARRAY System (Agena Bioscience Inc.) according to the manufacturer’s protocol. To ensure high-quality genotyping, quality control filters were used to remove SNPs with call rate <90%, samples with call rates <90%, and SNPs with a significant departure from Hardy-Weinberg equilibrium (p < 1E-04) resulting in 41 ieQTLs for analysis. Additionally, rare SNPs (defined by minor allele frequency <0.05) were removed prior to logistic regression analysis.

### Statistical analysis

Multivariate logistic regression genetic association analyses were conducted, adjusting for age at pathological diagnosis, sex (male vs. female), and Ashkenazi Jewish (AJ) status (yes vs. no), as a fraction of the patient cohort are of AJ ancestry (n = 281, 13.9%) (see Table 1). The logistic regression models were conducted comparing patients with MPM and no family history versus cancer-free controls, and those with MPM and no family history versus SPM and no family history. Because the GWAS controls did not provide AJ ancestry information, we performed Principal Component Analysis (PCA) on the control samples in order to identify any control samples of potential AJ ancestry, and then utilized this information to adjust all models for possible confounding due to AJ status. Additionally, we used whole genome sequencing (WGS) information obtained in our parallel ongoing studies for 49 of our AJ samples in this cohort to confirm the accuracy of self-reported AJ status in our clinical dataset. The PCA was done by first identifying overlapping variants among previously published AJ reference population^30^, AJ patients with WGS information, the GWAS control SNPs^14^, and genetic information from 1000 Genomes Phase 3, which served as a European population control. The 243,847 overlapping variants were pruned to a reduced set in linkage equilibrium using PLINK v1.90beta with parameters set to remove variants showing correlation r^2^ > 0.2 in 500-variant windows, and finally, the non-autosomal variants were removed. The remaining 97,000 variants were used for PCA, and putative AJ status was determined by samples with principal component 1 in the interval [−0.005, −0.00375] and principal component 2 in the interval [0.00375, 0.00625].

**Table 1:** Patient population characteristics.

Variable	Cases			Controls
	Overall Case Cohort (N = 977)	Multiple Melanomas (N = 147)	Single Primary Melanoma (N = 830)	Melanoma GWAS (N = 1047)*
**Age at primary diagnosis, N (%)**				
≤60	495 (50.7)	50 (34.0)	445 (53.6)	805 (76.9)
>60	482 (49.3)	97 (66.0)	385 (46.4)	242 (23.1)
**Gender, N (%)**				
Female	410 (42.0)	61 (42.0)	349 (42.0)	425 (40.6)
Male	567 (58.0)	86 (58.5)	481 (58.0)	622 (59.4)
**Ashkenazi ancestry, N (%)**				
Yes	243 (24.9)	41 (27.9)	202 (24.3)	38 (3.6)
No	734 (75.3)	106 (72.1)	628 (75.7)	1009 (96.4)
**Stage at primary diagnosis, N (%)**				
I	654 (66.9)	106 (72.1)	548 (66.0)	
II	159 (16.3)	21 (14.3)	138 (16.6)	
III	164 (16.8)	20 (13.6)	144 (17.3)	
**Immunotherapy Treatment, N (%)**				
No	898 (91.9)	141 (95.9)	757 (91.2)	
Yes	79 (8.1)	6 (4.1)	73 (8.8)	

*Ascertained at MD Anderson (phs000187.v1.p1).

Multivariate Cox proportional hazards (Cox PH) models were utilized to assess the association between SNPs and overall survival (OS) among either patients with MPM and no family history or patients with SPM and no family history of melanoma. Cox PH models were calculated from the age of diagnosis of the primary tumor until last follow-up, death, or treatment with immunotherapies and adjusted for age at pathological diagnosis, AJ status, sex (male vs. female), primary tumor histologic subtype (superficial-spreading vs. nodular vs. desmoplastic vs. acral-lentiginous vs. lentigo-maligna vs. other), and stage (I, II, III). MPM patients in this analysis were defined as those with 1 or more additional primary melanoma observed at initial diagnosis or developed subsequently after primary tumor diagnosis. Kaplan-Meier plots were produced for SNPs that reached significance after adjustment for multiple comparisons. Additive models of disease were used for the main effect logistic regression analysis, and both dominant and recessive models were selected for the survival analysis. Participants who received immunotherapy (N = 79) were censored at date of treatment. The adjustment for multiple testing was performed by Bonferroni procedure, which was employed in both logistic and survival models. Proportionality of hazards was assessed for all Cox models. All statistical analyses were conducted using PLINK^31^ and the “Survival” package in R.

### Ethical approval and informed consent

Written informed consents for the use of the blood specimens and clinical information were obtained at the time of enrollment from all participants, and the study was approved by the Institutional Review Board (IRB) at all NYULC (IRB#10362).

## Results

### Study population

The study population stems from a cohort of patients with stage I-III cutaneous melanoma who were ascertained as part of The Interdisciplinary Melanoma Collaboration Group (IMCG) at NYU Langone Health, as previously described^24–26^. The patients with family history of melanoma were specifically excluded from the analysis to reduce any confounding effects of known germline genetic factors associated with MPM or SPM development. 49.3% of patients were over 60 years of age and all patients were self-reported to be of European descent. The overall 5-year survival was 84.1% and the median time between diagnosis and follow up was 52.7 months. The majority of primary diagnoses were stage I and II (83%), and the most common histological subtype was superficial spreading melanoma (46.5%) followed by nodular melanoma (23.1%) and other subtypes (30.4%). The study population had no reported family history of melanoma and consists of 147 patients with multiple primary melanomas and 830 patients with single primary melanomas. For the case/control analyses, we have utilized data from 1047 control samples ascertained as part of a Genome Wide Association Study (GWAS) (phs000187.v1.p1)^14^. The clinical and demographic data for the patient population used in the current analyses are summarized in Table 1.

### The stratification of Ashkenazi Jewish (AJ) ancestry in case/control populations

A fraction of the patient population used in this analysis is of self-reported Ashkenazi Jewish (AJ) descent (n = 243, 24.9%); therefore, we ensured the stratification of AJ ancestry was appropriately adjusted. Also, the AJ ancestry status was not available for the GWAS control data. To validate the accuracy of self-reported AJ status information in IMCG cohort, we have exploited the whole genome sequencing (WGS) data available for a subset of 49 melanoma cases, sequenced as part of our parallel efforts and also used in the current analysis. Using WGS data from 49 self-reported AJ participants, the GWAS data of n = 128 AJ references samples genotyped as part of our prior study^30^, the GWAS control data^14^, and the genotyping data from the 1000 Genomes Project Phase 3, we identified 243,847 SNPs overlapping with the cancer-free GWAS control dataset. After linkage disequilibrium pruning, we performed principal component analysis (PCA) on 97,000 SNPs and plotted the two major principal components. 49 samples self-reported as AJ and 38 cancer-free GWAS control samples clustered around the AJ reference set. The remaining case and control samples spread among the different European sub-populations (Supplementary Fig. 1) as expected. For all further analyses in this study, we considered the controls clustering in the AJ reference set to be of AJ ancestry along with those of self-reported AJ ancestry in our clinical IMCG dataset.

### The association of ieQTLs with MPM risk

**Figure 1:**
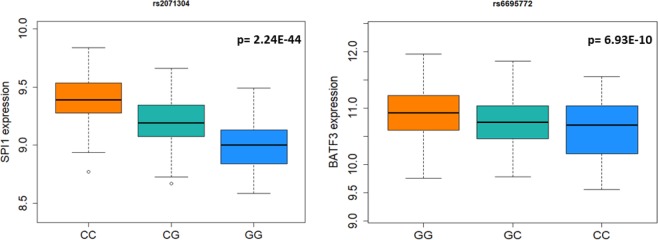
Genotype/gene expression correlation for the variants most significantly associated with MPM risk and MPM survival. The correlation between the genotype and gene expression level in LCLs along with the statistical significance (Linear mixed model p-value) were obtained from the MuTHER data^23^. Each genotype was plotted, with reference allele genotypes on the right of each graph. rs2071304 (SPI1) (left plot) is associated with MPM risk and rs6695772 (BATF3) (right plot) is associated with survival.

To explore MPM-specific associations, we performed an association analysis comparing MPM and SPM cases under an additive logistic regression model adjusting for age, sex and AJ status. The most significant association, surpassing the adjustment for multiple testing (Bonferroni threshold p < 0.001), was found for rs2071304 (OR = 0.60; 95% CI = 0.45–0.81; p = 0.0007), (Table 2, full results Supplementary Table 1). In order to test whether the most significant associations from this analysis were MPM-specific, we analyzed rs2071304 and rs665241 using two association tests comparing 147 MPM or 830 SPM, respectively, with 1047 healthy controls from publicly available imputed GWAS data (phs000187.v1.p1)^14^, adjusting for age, sex, and AJ status (for controls detected by PCA as described in Materials and Methods) (Table 2, full results Supplementary Tables 2 and 3). While no significant associations have been found in SPM case/control comparison, the associations were confirmed, albeit with reduced significance, for rs2071304 in MPM case/control analysis (OR = 0.59; 95% CI = 0.41–0.83; p = 0.0025). Again, the results showed that the alternate allele G of rs2071304, which associates with decreased expression of *SPI1* in LCLs (Fig. 1) in the MuTHER data^23^, confers a protective effect for MPM. It is possible that the results may be confounded by time of follow-up for SPM patients, biasing potential future diagnosis of MPM. To address this, for the 2 most significant variants from the initial analysis (rs2071304 and rs665241) we have also performed a subset analysis comparing MPM patients to those with SPM with at least 8 years of follow-up, a threshold established in prior studies^32^. Logistic regression was performed under the additive model adjusting for age, sex, and AJ status, comparing 147 MPM cases versus 142 SPM cases with at least 8 years follow up of single primary diagnosis. For both rs665241 and rs2071304, the associations were still significant in this more stringent analysis (OR = 0.58; 95% CI = 0.39–0.87; p = 0.0081; OR = 0.58; 95% CI = 0.40–0.83; p = 0.0031, respectively) (Supplementary Table 4).

**Table 2:** Summary of the most significant associations of immunomodulatory ieQTLs with MPMs when compared to SPMs and/or disease-free controls, under the additive model (adjusted for age at pathological diagnosis, sex (male vs. female), and Ashkenazi Jewish status (yes vs. no)).

SNP	Gene	SNP Position (GRCh38.p12)	Alternate Allele in the Population	Alternate allele frequency MPM patients	Alternate allele frequency SPM patients	Alternate allele frequency disease free controls	MPM vs SPM		MPM vs disease-free controls*	
							OR (95% C.I.)	*p*-value	OR (95% C.I.)	*p*-value
rs2071304	SPI1	chr11:47350826	G	0.25	0.35	0.32	0.60 (0.45, 0.81)	0.0007	0.59 (0.41, 0.83)	0.0025
rs665241	FYB	chr5:39266460	C	0.4	0.47	0.42	0.71 (0.55, 0.93)	0.0111	0.89 (0.63, 1.13)	0.2141
rs7720838	PTGER4	chr5:40486794	G	0.38	0.42	0.43	1.23 (0.94, 1.60)	0.1313	0.66 (0.49, 0.89)	0.0058
rs13331952	CKLF	chr16:66549715	C	0.09	0.12	0.13	0.76 (0.50, 1.17)	0.2177	0.59 (0.34, 0.93)	0.0231
rs9895554	SKAP1	chr17:48046280	C	0.1	0.09	0.07	1.15 (0.74, 1.77)	0.5355	1.69 (1.02, 2.63)	0.0389
rs9863627	PAK2	chr3:196808928	G	0.09	0.1	0.15	1.15 (0.73, 1.82)	0.5358	0.54 (0.33, 0.87)	0.0111
rs2276645	ZAP70	chr2:97713589	T	0.31	0.34	0.44	0.95 (0.71, 1.28)	0.7496	0.69 (0.49, 0.83)	0.0083

*Disease-free controls were ascertained at MD Anderson (phs000187.v1.p1)^14^.

Results passing the adjustments for multiple testing are highlighted in bold.

### ieQTLs associated with OS of patients with multiple melanomas

**Figure 2:**
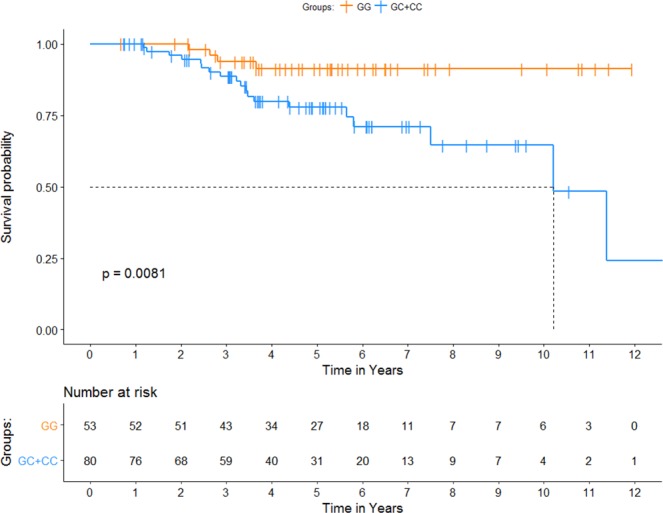
Kaplan-Meier plot of overall survival by BATF3 genotypes. KM curves of MPM survival for rs6695772 (*BATF3*). The carriers of the alternate allele (C) show significantly worse OS. Survival curves were generated using univariate Kaplan-Meier estimates. P-values were estimated using log-rank test.

We have previously reported that ieQTLs may be clinically actionable prognostic biomarkers predictive of OS among melanoma patients in the general population regardless of their MPM status^25^. Based on this suggestive evidence, we sought to test whether ieQTLs may also predict survival among patients with MPM. We performed Cox PH analysis on 147 MPM cases genotyped for 41 ieQTLs in this study. The results of the additive and dominant Cox proportional hazards model analyses are summarized in Table 3 (full results Supplementary Table 5). The most significant variant associated with MPM survival was rs6695772 (additive model: HR = 3.42; 95% CI = 1.57–7.42; p = 0.0019; dominant model: HR = 18.69; 95% CI = 3.34–104.55; p = 0.0009) (Fig. 2), linked to the expression of *BATF3*, which was the same ieQTL predictive of melanoma survival in our recent study^25^, with the alternate allele of rs6695772 linked to the decreased expression of *BATF*3 in LCLs (Fig. 1). While the effect of this variant observed in SPM patients was only of borderline significance (dominant model: HR = 1.65; 95% CI = 1.08–2.50; p = 0.0197) (Supplementary Table 6), the pooled analysis of both SPM and MPM cases showed association with rs6695772 and survival (dominant model: HR = 1.83; 95% CI = 1.25–2.69; p = 0.0021), suggesting that the observed association effect is predominantly driven by MPM cases. Using the univariate analysis (no adjustments for stage and tumor location), the associations of rs6695772 with survival remained comparably significant (additive model: HR = 2.16; 95% CI = 1.22–3.83; p = 0.0085, dominant model: HR = 4.85; 95% CI = 1.63–14.45; p = 0.0045).

**Table 3:** Summary of the most significant associations of immunomodulatory ieQTLs with overall survival among MPMs, under the additive and dominant models (adjusted for age at pathological diagnosis, sex (male vs. female), Ashkenazi Jewish status (yes vs. no), primary tumor histologic subtype (superficial-spreading vs. nodular vs. desmoplastic vs. acral-lentiginous vs. lentigo-maligna vs. other), and AJCC staging at diagnosis).

SNP	Gene	SNP Position (GRCh38.p12)	Alternate Allele in the population	Alternate allele frequency MPM patients	Hazard Ratio (95% C.I.)	p-value	Hazard Ratio (95% C.I.)	p-value
					Additive Model		Dominant Model	
rs6695772	BATF3	chr1:212708597	C	0.36	3.42 (1.57, 7.42)	**0.0019**	18.69 (3.34, 104.55)	**0.0009**
rs2291299	CCL5	chr17:35864402	G	0.18	0.14 (0.03, 0.66)	0.0133	0.14 (0.03, 0.66)	0.0133
rs12401573	SEMA4A	chr1:156176427	C	0.40	1.80 (0.93, 3.50)	0.0824	3.77 (1.22, 11.67)	0.0213
rs4500045	PAG1	chr8:81105697	A	0.51	2.45 (1.20, 5.02)	0.0142	3.24 (0.89, 11.85)	0.075
rs841718	STAT6	chr12:57099213	C	0.42	2.16 (1.11, 4.21)	0.0232	2.45 (0.86, 7.04)	0.0951

Top results are highlighted in bold.

## Discussion

A role of host immunity in controlling melanoma progression has been recently demonstrated by the successes of melanoma immune checkpoint blockade (ICB) therapies^33–35^. The ICB significantly improves survival of metastatic melanoma patients and there is a clear promise of ICB benefits at earlier stages as evidenced from ongoing adjuvant trials in melanoma^36^. These groundbreaking developments represent a paradigm showing that melanoma, an immunogenic tumor, attracts the attention of the host immune response which may be a modulating factor in tumor burden reduction. While this link has been clearly established in the context of advanced disease, it is highly plausible that the host immunity will also play a critical role in melanoma development. This is clearly supported by the studies showing that the risk of melanoma^37^, or tumor recurrence^9^ are increased among individuals who are immunosuppressed, through causes such as HIV infection^38,39^ or organ transplants. In this study, we tested a hypothesis that the genetic variants impacting host immune regulation may contribute to the development of multiple primary melanoma. We postulate that the immune surveillance of early stage primary tumors may be impaired by genetic factors, which in turn can be direct surrogate markers of additional primary tumor development. As we suggest, this may be a convenient clinical complement to the lengthy process of follow-up of patients with single primary melanomas, often undergoing many skin biopsies in order to reduce the likelihood of second melanoma, which is financially and emotionally burdensome. Thus, given the lifetime risk of additional primary melanomas ranging between 1–12%^40^, the availability of novel personalized biomarkers predictive of multiple primary tumors would be of significant clinical importance.

To address these assumptions, we analyzed 41 ieQTLs that associate with the expression of immune responsive genes in immune lymphopbasloid cells, from a healthy twin cohort in the MuTHER consortia^23,25^, in order to identify variants predictive of risk of MPM development. We have previously reported that such ieQTLs impact melanoma survival^25^. Here, we report significant associations of these 41 ieQTLs with the development of MPM. These findings suggest that immune modulation controlled by inherited genetic variants may be a contributing factor affecting the development of additional primary tumors in patients with single primary melanoma. The most significant result was found for rs2071304 (OR = 0.60; 95% CI = 0.45–0.81; p = 0.0007) comparing MPM patients with single primary cases. We found that patients who carried the alternate allele (G) in the study population are 40% less likely to develop MPM. In this analysis, the consideration of time to second primary from single primary diagnosis is critical, and as such we have also tested whether this association remained significant after accounting for sufficient follow-up time after initial SPM diagnosis. Importantly, rs2071304 remained among the top 2 most significant results after considering only SPM patients with at least 8 years of follow up after single primary diagnosis, an interval suggested in prior studies^32^. In addition, when we compared MPM samples with healthy controls, we found that rs2071304 showed borderline significance (p = 0.0025), which was not observed in the analysis of SPM patients versus healthy controls, further suggesting that the association is MPM-specific. While these findings suggest that rs2071304 may modulate risk of MPM, the data also raise a possibility that the risk alleles affecting the development of second primary melanomas from single primary diagnoses may not be necessarily identifiable by MPM case/control analysis. In controls, the risk alleles are not under selection pressure and are comparably distributed, as they do not associate with melanoma risk per se, but with the increased susceptibility to MPM, given the prior SPM diagnosis. This may explain attenuated significance of rs2071304 associations in comparison of MPM cases with healthy controls. Hence, the MPM-specific alleles may be fully identifiable only by comparison of MPM versus SPM patients, an analysis in which the observed associations reached the strongest significance in our study. The minor allele of rs2071304 was shown to correlate with decreased expression of *SPI1* in the MuTHER dataset (Fig. 1). *SPI1* has previously been shown to be involved in the development of several different types of immune lineage precursor cells, including, T-cells, B-cells, dendritic cells (DC) and monocytes^41–44^. Lowered levels of PU.1, the protein product of *SPI1*, have also been shown to result in preferential development of B1 B cells^45^, which are involved in innate immunity and are often autoreactive^46^, and other studies suggest that decreased levels of PU.1 associate with autoimmune conditions^47^. Given this evidence and the results of this study, the patients with the “low-expressing” SPI1 allele could potentially have increased sensitivity to self-antigens, what may allow for improved clearing of “micro” melanomas before they developed into detectable MPMs^48^. In addition to its role in the immune system, SPI1 has been shown to upregulate *Mcl-1* transcription in melanoma cells^49^ and this upregulation prevents melanoma cells from undergoing endoplasmic reticulum stress-induced apoptosis^50^. As rs2071304 is strongly associated with expression of SPI1 in skin cells (p < 1E-15) in the MuTHER data, with the same directionality as in LCLs, this is another potential mechanism explaining how the alternate allele of rs2071304 is protective against MPM.

Lastly, we tested if ieQTLs were able to predict survival in MPM patients. The most significant association with OS was found for rs6695772, showing that the minor allele (C) associates with significantly worse survival in MPM patients (additive model: HR = 3.42; 95% CI = 1.57–7.42; p = 0.0019, dominant model: HR = 18.69; 95% CI 3.34–104.55; p = 0.0009), although the relatively wide confidence interval suggests that this result needs to be validated in a cohort with larger statistical power. Interestingly, this association with OS was reported in our recent study in a general melanoma population^25^, in which we suggested that rs6695772 is a putative inherited risk marker predictive of melanoma survival. While only borderline association was observed in SPM patients in this study, the pooled analysis of SPM and MPM patients showed association effect of rs6695772 with survival, comparable with MPM analysis alone. Hence, while further validation in MPM and SPM patients will be needed to confirm MPM specific association of this variant with survival, the findings from the current study suggest that the effect of rs6695772 on survival is more pronounced in MPM patients. Noteworthy, the association observed in univariate analysis remained comparably significant, strongly suggesting that rs6695772 associates with OS independently of other clinical and prognostic predictors. We have also attempted to test this association with melanoma-specific survival. However, this comparison was underpowered due to the lack of melanoma-specific death information for most of the patients. Nevertheless, by restricting the analysis to melanoma-specific survival we have found that rs6695772 was still the most significant association with MPM, albeit with reduced significance level (HR = 11.27; 95% CI = 2.05–61.90; p = 0.0053). As we discussed extensively in our prior report^25^, this ieQTL associates with decreased expression of *BATF3* in LCLs in MuTHER dataset (Fig. 1). It has been documented that the loss of *BATF3* decreases the ability of dendritic cells in presenting cell-associated antigens via the MHC-I complex, thereby impairing baseline antitumor response^51^. As such the effect of “low expressing allele” at this locus associated with MPM survival may have significant implications for impaired immunity affecting melanoma progression. Given the findings in this study and our prior observations^25^, this region should be examined further in larger studies for its prognostic value in improving the current clinical outcome assessments in melanoma progression.

In summary, our study has identified several ieQTLs that associate with the development of multiple primary melanomas following the single primary diagnosis. In particular, rs2071304 showed the strongest association with MPMs compared to patients with SPM. The results of our study suggest that the potential modifications to the host immune response competency via an interplay of germline genetic factors, may be a crucial trigger in altering the likelihood of patients with primary melanomas developing subsequent primary tumors. Given that patients who develop additional primary melanomas are at a greater 10-year mortality risk compared to those with single primary tumors^3^, the genetic markers predictive of MPM development reported here, if validated, can potentially provide new opportunities for improving screening and clinical management of a high-risk population in which early identification has been challenging. While requiring further validation in large collaborative efforts, our results suggest that besides other pathological and clinical factors of progression from single primary tumors to multiple primary melanomas (tumor genetics, microenvironment, baseline immunity, etc.), additional research should also strongly consider germline genetic underpinning. As we emphasize, addition of germline genetic biomarkers identified in such efforts could have substantial clinical benefit for MPM high-risk patients as well as those in general melanoma population.

## Supplementary information

Supplementary Tables and Figures

## References

[1] Gupta BK, Piedmonte MR, Karakousis CP (1991). Attributes and survival patterns of multiple primary cutaneous malignant melanoma. Cancer.

[2] Leiter U (2012). Hazard rates for recurrent and secondary cutaneous melanoma: an analysis of 33,384 patients in the German Central Malignant Melanoma Registry. J Am Acad Dermatol.

[3] Youlden DR (2016). Ten-Year Survival after Multiple Invasive Melanomas Is Worse than after a Single Melanoma: a Population-Based Study. J Invest Dermatol.

[4] Hwa C (2012). Single versus multiple primary melanomas: old questions and new answers. Cancer.

[5] Ferrone CR (2005). Clinicopathological features of and risk factors for multiple primary melanomas. Jama-J Am Med Assoc.

[6] Slingluff CL, Vollmer RT, Seigler HF (1993). Multiple primary melanoma: incidence and risk factors in 283 patients. Surgery.

[7] Goggins WB, Tsao H (2003). A population-based analysis of risk factors for a second primary cutaneous melanoma among melanoma survivors. Cancer.

[8] Mayor Paul C., Eng Kevin H., Singel Kelly L., Abrams Scott I., Odunsi Kunle, Moysich Kirsten B., Fuleihan Ramsay, Garabedian Elizabeth, Lugar Patricia, Ochs Hans D., Bonilla Francisco A., Buckley Rebecca H., Sullivan Kathleen E., Ballas Zuhair K., Cunningham-Rundles Charlotte, Segal Brahm H. (2018). Cancer in primary immunodeficiency diseases: Cancer incidence in the United States Immune Deficiency Network Registry. Journal of Allergy and Clinical Immunology.

[9] Asgari MM, Ray GT, Quesenberry CP, Katz KA, Silverberg MJ (2017). Association of Multiple Primary Skin Cancers With Human Immunodeficiency Virus Infection, CD4 Count, and Viral Load. JAMA Dermatol.

[10] Zhang M (2013). Genome-wide association studies identify several new loci associated with pigmentation traits and skin cancer risk in European Americans. Human molecular genetics.

[11] Macgregor S (2011). Genome-wide association study identifies a new melanoma susceptibility locus at 1q21.3. Nature genetics.

[12] Barrett JH (2011). Genome-wide association study identifies three new melanoma susceptibility loci. Nature genetics.

[13] Bishop DT (2009). Genome-wide association study identifies three loci associated with melanoma risk. Nature genetics.

[14] Amos CI (2011). Genome-wide association study identifies novel loci predisposing to cutaneous melanoma. Human molecular genetics.

[15] Law MH, Macgregor S, Hayward NK (2012). Melanoma genetics: recent findings take us beyond well-traveled pathways. J Invest Dermatol.

[16] Millikan RC (2006). Polymorphisms in nucleotide excision repair genes and risk of multiple primary melanoma: the Genes Environment and Melanoma Study. Carcinogenesis.

[17] Gibbs DC (2015). Inherited genetic variants associated with occurrence of multiple primary melanoma. Cancer Epidemiol Biomarkers Prev.

[18] Hatvani Z (2014). Genotype analysis in Hungarian patients with multiple primary melanoma. Experimental dermatology.

[19] Helsing P (2012). MC1R, ASIP, TYR, and TYRP1 gene variants in a population-based series of multiple primary melanomas. Genes Chromosomes Cancer.

[20] Kanetsky PA (2006). Population-based study of natural variation in the melanocortin-1 receptor gene and melanoma. Cancer Res.

[21] Bruno W (2016). Multiple primary melanomas (MPMs) and criteria for genetic assessment: MultiMEL, a multicenter study of the Italian Melanoma Intergroup. J Am Acad Dermatol.

[22] Mandelcorn-Monson Rochelle, Marrett Loraine, Kricker Anne, Armstrong Bruce K., Orlow Irene, Goumas Chris, Paine Susan, Rosso Stefano, Thomas Nancy, Millikan Robert C., Pole Jason D., Cotignola Javier, Rosen Cheryl, Kanetsky Peter A., Lee-Taylor Julia, Begg Colin B., Berwick Marianne (2011). Sun exposure, vitamin D receptor polymorphisms FokI and BsmI and risk of multiple primary melanoma. Cancer Epidemiology.

[23] Nica AC (2011). The architecture of gene regulatory variation across multiple human tissues: the MuTHER study. PLoS Genet.

[24] Rendleman J (2013). Melanoma risk loci as determinants of melanoma recurrence and survival. Journal of translational medicine.

[25] Vogelsang M (2016). The Expression Quantitative Trait Loci in Immune Pathways and their Effect on Cutaneous Melanoma Prognosis. Clinical cancer research: an official journal of the American Association for Cancer Research.

[26] Rendleman J (2015). Genetic associations of the interleukin locus at 1q32.1 with clinical outcomes of cutaneous melanoma. J Med Genet.

[27] Qian M (2013). Clinicopathological characteristics at primary melanoma diagnosis as risk factors for brain metastasis. Melanoma research.

[28] Grundberg E (2012). Mapping cis- and trans-regulatory effects across multiple tissues in twins. Nature genetics.

[29] Spector TD, Williams FM (2006). The UK Adult Twin Registry (TwinsUK). Twin research and human genetics: the official journal of the International Society for Twin Studies.

[30] Vijai J (2013). Susceptibility loci associated with specific and shared subtypes of lymphoid malignancies. PLoS Genet.

[31] Purcell S (2007). PLINK: a tool set for whole-genome association and population-based linkage analyses. American journal of human genetics.

[32] Jones MS (2016). Second Primary Melanoma: Risk Factors, Histopathologic Features, Survival, and Implications for Follow-Up. Am Surgeon.

[33] Hodi FS (2010). Improved survival with ipilimumab in patients with metastatic melanoma. N Engl J Med.

[34] Robert C (2015). Pembrolizumab versus Ipilimumab in Advanced Melanoma. N Engl J Med.

[35] Robert C (2015). Nivolumab in Previously Untreated Melanoma without BRAF Mutation. New Engl J Med.

[36] Weber J (2017). Adjuvant Nivolumab versus Ipilimumab in Resected Stage III or IV Melanoma. N Engl J Med.

[37] Kubica AW, Brewer JD (2012). Melanoma in immunosuppressed patients. Mayo Clin Proc.

[38] Patel P (2008). Incidence of types of cancer among HIV-infected persons compared with the general population in the United States, 1992-2003. Ann Intern Med.

[39] Wilkins Karl, Turner Ryan, Dolev Jacqueline C., LeBoit Philip E., Berger Timothy G., Maurer Toby A. (2006). Cutaneous malignancy and human immunodeficiency virus disease. Journal of the American Academy of Dermatology.

[40] Juul Nielsen L, Rosenkrantz Holmich L (2016). Eleven Primary Melanomas, Colon Cancer, and Atypical Nevi in the Same Patient: A Case Report and Literature Review. Case Rep Dermatol Med.

[41] Scott EW, Simon MC, Anastasi J, Singh H (1994). Requirement of transcription factor PU.1 in the development of multiple hematopoietic lineages. Science.

[42] McKercher SR (1996). Targeted disruption of the PU.1 gene results in multiple hematopoietic abnormalities. EMBO J.

[43] Anderson KL (2000). Transcription factor PU.1 is necessary for development of thymic and myeloid progenitor-derived dendritic cells. J Immunol.

[44] Merad M, Sathe P, Helft J, Miller J, Mortha A (2013). The dendritic cell lineage: ontogeny and function of dendritic cells and their subsets in the steady state and the inflamed setting. Annu Rev Immunol.

[45] Rosenbauer F (2006). Lymphoid cell growth and transformation are suppressed by a key regulatory element of the gene encoding PU.1. Nature genetics.

[46] Kaveri SV, Silverman GJ, Bayry J (2012). Natural IgM in Immune Equilibrium and Harnessing Their Therapeutic Potential. Journal of Immunology.

[47] Alivernini, S. *et al*. MicroRNA-155 influences B-cell function through PU.1 in rheumatoid arthritis. *Nat Commun* **7**, 10.1038/ncomms12970 (2016). 10.1038/ncomms12970 PMC5052655 27671860

[48] Mahmoud F (2017). Immune surveillance in melanoma: From immune attack to melanoma escape and even counterattack. Cancer Biol Ther.

[49] Wang, J. M., Lai, M. Z. & Yang-Yen, H. F. Interleukin-3 stimulation of mcl-1 gene transcription involves activation of the PU.1 transcription factor through a p38 mitogen-activated protein kinase-dependent pathway. *Mol Cell Biol* **23**, 1896-1909 (2003). 10.1128/MCB.23.6.1896-1909.2003 PMC149468 12612065

[50] Jiang CC (2008). Up-regulation of Mcl-1 is critical for survival of human melanoma cells upon endoplasmic reticulum stress. Cancer Res.

[51] Hildner K (2008). Batf3 deficiency reveals a critical role for CD8alpha+ dendritic cells in cytotoxic T cell immunity. Science.

