# Supplementary material for: Immunomodulatory germline variation associated with the development of multiple primary melanoma (MPM)
Source: Sci Rep. 2019 Jul 15;9:10173. doi: 10.1038/s41598-019-46665-z (PMC6629847; doi:10.1038/s41598-019-46665-z)
Supplement: Supplementary file 1 — Supplementary Tables and Figures [file 41598_2019_46665_MOESM1_ESM.pdf]

Immunomodulatory germline variation associated with the development of multiple primary melanoma (MPM)

Robert Ferguson[1,2,3*], Alexi Archambault[1,2,3*], Danny Simpson[1,2,3], Leah Morales[1,2,3], Vylyny Chat[1,2,3], Esther Kazlow[1,2,3], Rebecca Lax[1,2,3], Garrett Yoon[1,2,3], Una Moran[1,3,4,5], Richard Shapiro[3,6], Anna Pavlick[3,4], David Polsky[1,3,5,7], Iman Osman[1,3,4,5], Tomas Kirchhoff[1,2,3**]

[1]Perlmutter Cancer Center, New York University School of Medicine, New York, USA
[2]Departments of Population Health and Environmental Medicine, New York University School of Medicine, New York, USA
[3]The Interdisciplinary Melanoma Cooperative Group, New York University School of Medicine, New York, USA
[4]Department of Medicine, New York University School of Medicine, New York, USA
[5]Ronald O. Perelman, Department of Dermatology, New York University, New York, USA
[6]Department of Surgery, New York University School of Medicine, New York, USA
[7]Department of Pathology, New York University School of Medicine, New York, USA
*These authors contributed equally to the work
** Corresponding author. Telephone: 2122639418. Email: Tomas.Kirchhoff@nyulangone.org

SUPPLEMENTARY TABLES AND FIGURES

Supplemental Table 1: Variant associations for MPMs when compared to SPMs, under the additive model*

SNP	Gene	SNP Position (GRCh38.p12)	Alternate Allele in our Population	Alternate allele frequency MPM patients	Alternate allele frequency SPM patients	OR (95% C.I.)	*p*-value
rs2071304	SPI1	chr11:47350826	G	0.25	0.35	0.60 (0.45, 0.81)	**0.0007**
rs665241	FYB	chr5:39266460	C	0.40	0.47	0.71 (0.55, 0.93)	0.0111
rs7584870	SOCS5	chr2:46746261	A	0.43	0.37	1.29 (0.99, 1.68)	0.055
rs8101605	LILRB1	chr19:54637036	A	0.21	0.16	1.32 (0.95, 1.81)	0.0948
rs7720838	PTGER4	chr5:40486794	G	0.38	0.42	1.23 (0.94, 1.60)	0.1313
rs859	IL16	chr15:81308981	G	0.23	0.28	0.80 (0.59, 1.07)	0.132
rs11161590	BCL10	chr1:85299315	G	0.41	0.45	0.82 (0.63, 1.06)	0.1344
rs4577037	IL16	chr15:81304319	G	0.05	0.08	0.66 (0.38, 1.14)	0.1353
rs7574070	STAT4	chr2:191145762	A	0.32	0.36	1.22 (0.92, 1.60)	0.1648
rs4848306	IL1B	chr2:112840530	A	0.45	0.41	1.19 (0.92, 1.54)	0.1857
rs13331952	CKLF	chr16:66549715	C	0.09	0.12	0.76 (0.50, 1.17)	0.2177
rs11569345	CD40	chr20:46129882	A	0.04	0.03	1.49 (0.78, 2.84)	0.2303
rs17001247	CXCL10	chr4:75949138	C	0.26	0.23	1.19 (0.89, 1.59)	0.2407
rs4796105	CCL5	chr17:35758948	C	0.12	0.15	0.80 (0.55, 1.18)	0.2623
rs1378940	CSK	chr15:74791153	G	0.36	0.39	0.86 (0.66, 1.13)	0.2743
rs6695772	BATF3	chr1:212708597	C	0.36	0.39	1.15 (0.89, 1.48)	0.298
rs3807383	GIMAP5	chr7:150737070	C	0.28	0.25	1.14 (0.86, 1.52)	0.3706
rs152112	ITK	chr5:157217613	T	0.28	0.31	0.88 (0.67, 1.18)	0.3958
rs1049337	CAV1	chr7:116560533	T	0.26	0.28	0.89 (0.65, 1.21)	0.4584
rs11203203	UBASH3A	chr21:42416077	A	0.40	0.42	0.91 (0.69, 1.18)	0.4672
rs9895554	SKAP1	chr17:48046280	C	0.10	0.09	1.15 (0.74, 1.77)	0.5355
rs9863627	PAK2	chr3:196808928	G	0.09	0.10	1.15 (0.73, 1.82)	0.5358
rs4808137	UBA52	chr19:18578037	A	0.50	0.47	0.93 (0.72, 1.21)	0.593
rs12401573	SEMA4A	chr1:156176427	C	0.40	0.42	1.07 (0.83, 1.37)	0.6217
rs2295359	IL23R	chr1:67170267	A	0.30	0.32	0.93 (0.70, 1.24)	0.632
rs11919943	CCR1	chr3:46225235	C	0.09	0.10	0.90 (0.58, 1.42)	0.6565
rs6692729	PSEN2	chr1:226831218	T	0.44	0.43	1.06 (0.82, 1.37)	0.6649
rs7036417	SYK	chr9:90808223	T	0.35	0.34	1.05 (0.80, 1.37)	0.7373

rs2276645	ZAP70	chr2:97713589	T	0.31	0.34	0.95 (0.71, 1.28)	0.7496
rs10422141	TICAM1	chr19:4833398	T	0.49	0.47	1.04 (0.80, 1.37)	0.7525
rs4500045	PAG1	chr8:81105697	A	0.51	0.49	1.04 (0.80, 1.34)	0.7921
rs2291299	CCL5	chr17:35864402	G	0.18	0.18	0.96 (0.68, 1.34)	0.7943
rs2701652	IRAK3	chr12:66187097	C	0.27	0.28	1.04 (0.78, 1.38)	0.8069
rs10760142	C5	chr9:121073276	C	0.46	0.45	1.03 (0.79, 1.33)	0.84
rs9921791	MLST8	chr16:2200067	T	0.08	0.08	1.05 (0.65, 1.68)	0.8562
rs1551565	CAMK4	chr5:111225941	C	0.26	0.26	1.02 (0.77, 1.37)	0.877
rs4402765	IL1A	chr2:112811270	C	0.27	0.27	0.98 (0.73, 1.32)	0.8983
rs4469949	CD27	chr12:6436445	A	0.38	0.38	1.01 (0.78, 1.31)	0.9467
rs841718	STAT6	chr12:57099213	C	0.42	0.41	0.99 (0.76, 1.30)	0.9643
rs6673928	IL19	chr1:206763900	T	0.23	0.23	1.01 (0.74, 1.36)	0.9702
rs1149901	GATA3	chr10:8052824	T	0.21	0.21	1.00 (0.73, 1.36)	0.9738

*Model adjusted for age at pathological diagnosis, sex (male vs. female), and Ashkenazi Jewish status (yes vs. no)

Supplemental Table 2: Variant associations for MPMs when compared to disease-free controls, under the additive model*

SNP	Gene	SNP Position (GRCh38.p12)	Alternate Allele in our Population	Alternate allele frequency MPM patients	Alternate allele frequency disease free controls	OR (95% C.I.)	*p*-value
rs2071304	SPI1	chr11:47350826	G	0.25	0.32	0.59 (0.41, 0.83)	**0.0025**
rs7720838	PTGER4	chr5:40486794	G	0.38	0.43	0.66 (0.49, 0.89)	0.0058
rs2276645	ZAP70	chr2:97713589	T	0.31	0.44	0.69 (0.49, 0.83)	0.0083
rs9863627	PAK2	chr3:196808928	G	0.09	0.15	0.54 (0.33, 0.87)	0.0111
rs13331952	CKLF	chr16:66549715	C	0.09	0.13	0.59 (0.34, 0.93)	0.0231
rs9895554	SKAP1	chr17:48046280	C	0.10	0.07	1.69 (1.02, 2.63)	0.0389
rs4796105	CCL5	chr17:35758948	C	0.12	0.18	0.69 (0.44, 1.03)	0.0593
rs4500045	PAG1	chr8:81105697	A	0.51	0.43	1.29 (0.96, 1.73)	0.0811
rs4808137	UBA52	chr19:18578037	A	0.50	0.42	1.27 (0.97, 1.67)	0.0864
rs8101605	LILRB1	chr19:54637036	A	0.21	0.16	1.29 (0.92, 1.83)	0.1341
rs841718	STAT6	chr12:57099213	C	0.42	0.37	1.29 (0.90, 1.63)	0.2006
rs4402765	IL1A	chr2:112811270	C	0.27	0.31	0.89 (0.59, 1.13)	0.204
rs11919943	CCR1	chr3:46225235	C	0.09	0.06	1.39 (0.82, 2.33)	0.2118
rs665241	FYB	chr5:39266460	C	0.40	0.42	0.89 (0.63, 1.13)	0.2141
rs1049337	CAV1	chr7:116560533	T	0.26	0.29	0.89 (0.60, 1.13)	0.2255
rs6692729	PSEN2	chr1:226831218	T	0.44	0.48	0.89 (0.66, 1.13)	0.3406
rs7574070	STAT4	chr2:191145762	A	0.32	0.36	0.87 (0.66, 1.16)	0.3435
rs9921791	MLST8	chr16:2200067	T	0.08	0.09	0.79 (0.46, 1.33)	0.3714
rs1149901	GATA3	chr10:8052824	T	0.21	0.26	0.86 (0.62, 1.20)	0.3833
rs4577037	IL16	chr15:81304319	G	0.05	0.08	0.79 (0.44, 1.33)	0.3997
rs11161590	BCL10	chr1:85299315	G	0.41	0.40	0.89 (0.66, 1.13)	0.4263
rs152112	ITK	chr5:157217613	T	0.28	0.31	0.89 (0.65, 1.23)	0.4274
rs4469949	CD27	chr12:6436445	A	0.38	0.35	1.19 (0.84, 1.43)	0.4373
rs11203203	UBASH3A	chr21:42416077	A	0.40	0.37	1.19 (0.83, 1.43)	0.4445
rs12401573	SEMA4A	chr1:156176427	C	0.40	0.39	1.11 (0.83, 1.48)	0.4735
rs4848306	IL1B	chr2:112840530	A	0.45	0.46	1.09 (0.83, 1.43)	0.5154
rs6673928	IL19	chr1:206763900	T	0.23	0.27	0.89 (0.65, 1.23)	0.5167
rs2295359	IL23R	chr1:67170267	A	0.30	0.34	0.99 (0.66, 1.23)	0.57
rs7584870	SOCS5	chr2:46746261	A	0.43	0.40	1.09 (0.81, 1.43)	0.5792

rs859	IL16	chr15:81308981	G	0.23	0.26	0.99 (0.66, 1.23)	0.5979
rs10760142	C5	chr9:121073276	C	0.46	0.46	0.99 (0.70, 1.23)	0.6077
rs7036417	SYK	chr9:90808223	T	0.35	0.35	1.09 (0.78, 1.43)	0.6784
rs10422141	TICAM1	chr19:4833398	T	0.49	0.44	1.09 (0.79, 1.43)	0.6882
rs1551565	CAMK4	chr5:111225941	C	0.26	0.27	0.99 (0.67, 1.23)	0.6899
rs17001247	CXCL10	chr4:75949138	C	0.26	0.25	0.99 (0.69, 1.23)	0.7281
rs1378940	CSK	chr15:74791153	G	0.36	0.34	0.99 (0.70, 1.23)	0.746
rs6695772	BATF3	chr1:212708597	C	0.36	0.33	1.03 (0.77, 1.39)	0.8243
rs11569345	CD40	chr20:46129882	A	0.04	0.04	1.09 (0.56, 2.03)	0.8442
rs2291299	CCL5	chr17:35864402	G	0.18	0.18	1.09 (0.71, 1.43)	0.8942
rs2701652	IRAK3	chr12:66187097	C	0.27	0.28	1.02 (0.75, 1.39)	0.8947
rs3807383	GIMAP5	chr7:150737070	C	0.28	0.28	1.09 (0.74, 1.33)	0.9468

*Model adjusted for age at pathological diagnosis, sex (male vs. female), and Ashkenazi Jewish status (yes vs. no)

Supplemental Table 3: Variant associations for SPMs when compared to disease-free controls, under the additive model*

SNP	Gene	SNP Position (GRCh38.p12)	Alternate Allele in our Population	Alternate allele frequency SPM patients	Alternate allele frequency disease free controls	OR (95% C.I.)	*p*-value
rs11919943	CCR1	chr3:46225235	C	0.10	0.06	1.65 (1.27,2.14)	**0.0002**
rs2276645	ZAP70	chr2:97713589	T	0.34	0.44	0.77 (0.66,0.89)	0.0003
rs1149901	GATA3	chr10:8052824	T	0.21	0.26	0.78 (0.66, 0.91)	0.0023
rs9863627	PAK2	chr3:196808928	G	0.10	0.15	0.72 (0.58, 0.89)	0.0027
rs4500045	PAG1	chr8:81105697	A	0.49	0.43	1.23 (1.07,1.41)	0.0036
rs4796105	CCL5	chr17:35758948	C	0.15	0.18	0.77 (0.64,0.93)	0.0059
rs11161590	BCL10	chr1:85299315	G	0.45	0.40	1.21 (1.06,1.39)	0.0064
rs6695772	BATF3	chr1:212708597	C	0.39	0.33	1.20 (1.05, 1.39)	0.0097
rs11203203	UBASH3A	chr21:42416077	A	0.42	0.37	1.19 (1.03,1.37)	0.0185
rs9895554	SKAP1	chr17:48046280	C	0.09	0.07	1.35 (1.04,1.74)	0.0232
rs841718	STAT6	chr12:57099213	C	0.41	0.37	1.17 (1.02,1.35)	0.0282
rs1378940	CSK	chr15:74791153	G	0.39	0.34	1.17 (1.01,1.35)	0.0316
rs2071304	SPI1	chr11:47350826	G	0.35	0.32	1.17 (1.01,1.36)	0.0347
rs4808137	UBA52	chr19:18578037	A	0.47	0.42	1.16 (1.01, 1.33)	0.0363
rs12401573	SEMA4A	chr1:156176427	C	0.42	0.39	1.15 (1.00, 1.32)	0.0487
rs17001247	CXCL10	chr4:75949138	C	0.23	0.25	0.86 (0.73,1.00)	0.0518
rs665241	FYB	chr5:39266460	C	0.47	0.42	1.15 (1.00,1.31)	0.052
rs4848306	IL1B	chr2:112840530	A	0.41	0.46	0.88 (0.76,1.00)	0.0548
rs7720838	PTGER4	chr5:40486794	G	0.42	0.43	0.87 (0.76, 1.01)	0.0594
rs6692729	PSEN2	chr1:226831218	T	0.43	0.48	0.88 (0.77,1.01)	0.0655
rs4402765	IL1A	chr2:112811270	C	0.27	0.31	0.87 (0.75,1.02)	0.0852
rs7584870	SOCS5	chr2:46746261	A	0.37	0.40	0.89 (0.77,1.02)	0.1024
rs6673928	IL19	chr1:206763900	T	0.23	0.27	0.88 (0.75,1.03)	0.103
rs8101605	LILRB1	chr19:54637036	A	0.16	0.16	0.87 (0.71,1.05)	0.1422
rs11569345	CD40	chr20:46129882	A	0.03	0.04	0.77 (0.55,1.10)	0.148
rs10760142	C5	chr9:121073276	C	0.45	0.46	0.90 (0.78,1.04)	0.1567
rs13331952	CKLF	chr16:66549715	C	0.12	0.13	0.86 (0.70,1.07)	0.182
rs4469949	CD27	chr12:6436445	A	0.38	0.35	1.10 (0.95,1.27)	0.189
rs3807383	GIMAP5	chr7:150737070	C	0.25	0.28	0.91 (0.78,1.06)	0.2285
rs9921791	MLST8	chr16:2200067	T	0.08	0.09	0.87 (0.68,1.12)	0.2742

rs152112	ITK	chr5:157217613	T	0.31	0.31	1.07 (0.92,1.24)	0.3826
rs4577037	IL16	chr15:81304319	G	0.08	0.08	0.90 (0.70,1.15)	0.3932
rs7574070	STAT4	chr2:191145762	A	0.36	0.36	0.94 (0.82, 1.09)	0.4341
rs2295359	IL23R	chr1:67170267	A	0.32	0.34	0.94 (0.81,1.09)	0.4358
rs1551565	CAMK4	chr5:111225941	C	0.26	0.27	0.94 (0.80,1.10)	0.4388
rs1049337	CAV1	chr7:116560533	T	0.28	0.29	0.94 (0.81,1.10)	0.4402
rs859	IL16	chr15:81308981	G	0.28	0.26	1.06 (0.91,1.23)	0.4775
rs2701652	IRAK3	chr12:66187097	C	0.28	0.28	1.03 (0.89, 1.20)	0.696
rs10422141	TICAM1	chr19:4833398	T	0.47	0.44	1.03 (0.89,1.18)	0.7209
rs7036417	SYK	chr9:90808223	T	0.34	0.35	0.98 (0.85,1.13)	0.764
rs2291299	CCL5	chr17:35864402	G	0.18	0.18	1.01 (0.85,1.20)	0.9306

*Model adjusted for age at pathological diagnosis, sex (male vs. female), and Ashkenazi Jewish status (yes vs. no)

Supplemental Table 4: Variant associations for MPMs when compared to SPMs with a minimum of 8 years follow-up data, under the additive model*

SNP	Gene	SNP Position (GRCh38.p12)	Alternate Allele in our Population	Alternate allele frequency MPM patients	Alternate allele frequency SPM patients	OR (95% C.I.)	*p*-value
rs665241	FYB	chr5:39266460	C	0.40	0.47	0.58 (0.40, 0.83)	**0.0031**
rs2071304	SPI1	chr11:47350826	G	0.25	0.35	0.58 (0.39, 0.87)	0.0081
rs9895554	SKAP1	chr17:48046280	C	0.10	0.09	2.05 (1.04, 4.02)	0.0378
rs13331952	CKLF	chr16:66549715	C	0.09	0.12	0.62 (0.36, 1.05)	0.0754
rs8101605	LILRB1	chr19:54637036	A	0.21	0.16	1.43 (0.91, 2.25)	0.1194
rs9863627	PAK2	chr3:196808928	G	0.09	0.10	0.64 (0.36, 1.15)	0.1338
rs1378940	CSK	chr15:74791153	G	0.36	0.39	0.76 (0.53, 1.09)	0.1347
rs4848306	IL1B	chr2:112840530	A	0.45	0.41	1.31 (0.91, 1.89)	0.1495
rs859	IL16	chr15:81308981	G	0.23	0.28	0.75 (0.50, 1.12)	0.1559
rs4500045	PAG1	chr8:81105697	A	0.51	0.49	1.29 (0.90, 1.84)	0.1605
rs7036417	SYK	chr9:90808223	T	0.35	0.34	0.78 (0.54, 1.13)	0.1883
rs1149901	GATA3	chr10:8052824	T	0.21	0.21	1.33 (0.86, 2.06)	0.2012
rs7574070	STAT4	chr2:191145762	A	0.32	0.36	0.80 (0.55, 1.15)	0.2286
rs7584870	SOCS5	chr2:46746261	A	0.43	0.37	1.25 (0.87, 1.79)	0.2343
rs4402765	IL1A	chr2:112811270	C	0.27	0.27	0.78 (0.52, 1.18)	0.2397
rs4577037	IL16	chr15:81304319	G	0.05	0.08	1.64 (0.72, 3.72)	0.2404
rs11203203	UBASH3A	chr21:42416077	A	0.40	0.42	0.81 (0.56, 1.19)	0.2866
rs6692729	PSEN2	chr1:226831218	T	0.44	0.43	1.18 (0.82, 1.70)	0.363
rs11919943	CCR1	chr3:46225235	C	0.09	0.10	0.76 (0.41, 1.39)	0.3721
rs10422141	TICAM1	chr19:4833398	T	0.49	0.47	1.17 (0.82, 1.67)	0.3845
rs11161590	BCL10	chr1:85299315	G	0.41	0.45	0.85 (0.59, 1.24)	0.405
rs2291299	CCL5	chr17:35864402	G	0.18	0.18	1.23 (0.74, 2.05)	0.4184
rs3807383	GIMAP5	chr7:150737070	C	0.28	0.25	1.19 (0.79, 1.79)	0.4184
rs9921791	MLST8	chr16:2200067	T	0.08	0.08	1.37 (0.64, 2.93)	0.4225
rs841718	STAT6	chr12:57099213	C	0.42	0.41	0.86 (0.60, 1.24)	0.4257
rs7720838	PTGER4	chr5:40486794	G	0.38	0.42	0.87 (0.59, 1.26)	0.4581
rs2276645	ZAP70	chr2:97713589	T	0.31	0.34	0.87 (0.58, 1.31)	0.5097
rs6695772	BATF3	chr1:212708597	C	0.36	0.39	1.11 (0.78, 1.59)	0.5573
rs1049337	CAV1	chr7:116560533	T	0.26	0.28	0.89 (0.58, 1.35)	0.5728

rs2295359	IL23R	chr1:67170267	A	0.30	0.32	0.91 (0.62, 1.33)	0.615
rs4796105	CCL5	chr17:35758948	C	0.12	0.15	0.89 (0.52, 1.53)	0.6754
rs4469949	CD27	chr12:6436445	A	0.38	0.38	1.07 (0.73, 1.55)	0.7411
rs152112	ITK	chr5:157217613	T	0.28	0.31	1.05 (0.69, 1.59)	0.8134
rs10760142	C5	chr9:121073276	C	0.46	0.45	0.97 (0.69, 1.35)	0.8386
rs6673928	IL19	chr1:206763900	T	0.23	0.23	1.04 (0.67, 1.62)	0.85
rs12401573	SEMA4A	chr1:156176427	C	0.40	0.42	0.97 (0.68, 1.38)	0.8743
rs4808137	UBA52	chr19:18578037	A	0.50	0.47	1.03 (0.72, 1.46)	0.8747
rs2701652	IRAK3	chr12:66187097	C	0.27	0.28	1.03 (0.70, 1.53)	0.8802
rs11569345	CD40	chr20:46129882	A	0.04	0.03	0.97 (0.40, 2.35)	0.9543
rs1551565	CAMK4	chr5:111225941	C	0.26	0.26	1.01 (0.68, 1.48)	0.971
rs17001247	CXCL10	chr4:75949138	C	0.26	0.23	1.00 (0.67, 1.50)	0.9954

*Model adjusted for age at pathological diagnosis, sex (male vs. female), and Ashkenazi Jewish status (yes vs. no)

Supplemental Table 5: Variant associations for survival among MPMs, under the additive and dominant models*

SNP	Gene	SNP Position (GRCh38.p12)	Alternate Allele in our Population	Alternate allele frequency MPM patients	Hazard Ratio (95% C.I.)	*p*-value	Hazard Ratio (95% C.I.)	*p*-value
					Additive Model		*Dominant Model*	
rs6695772	BATF3	chr1:212708597	C	0.36	3.42 (1.57, 7.42)	**0.0019**	18.69 (3.34, 104.55)	**0.0009**
rs2291299	CCL5	chr17:35864402	G	0.18	0.14 (0.03, 0.66)	0.0133	0.14 (0.03, 0.66)	0.0133
rs12401573	SEMA4A	chr1:156176427	C	0.40	1.80 (0.93, 3.50)	0.0824	3.77 (1.22, 11.67)	0.0213
rs4500045	PAG1	chr8:81105697	A	0.51	2.45 (1.20, 5.02)	0.0142	3.24 (0.89, 11.85)	0.075
rs13331952	CKLF	chr16:66549715	C	0.09	0.22 (0.04, 1.20)	0.0806	0.21 (0.04, 1.18)	0.0764
rs9921791	MLST8	chr16:2200067	T	0.08	2.51 (0.90, 7.03)	0.0789	2.51 (0.90, 7.03)	0.0789
rs3807383	GIMAP5	chr7:150737070	C	0.28	0.46 (0.20, 1.07)	0.0701	0.42 (0.16, 1.14)	0.0877
rs859	IL16	chr15:81308981	G	0.23	0.67 (0.29, 1.55)	0.3528	0.41 (0.15, 1.15)	0.0895
rs841718	STAT6	chr12:57099213	C	0.42	2.16 (1.11, 4.21)	0.0232	2.45 (0.86, 7.04)	0.0951
rs11569345	CD40	chr20:46129882	A	0.04	0.14 (0.01, 1.42)	0.0963	0.14 (0.01, 1.42)	0.0963
rs4796105	CCL5	chr17:35758948	C	0.12	0.29 (0.06, 1.30)	0.105	0.29 (0.06, 1.30)	0.105
rs2701652	IRAK3	chr12:66187097	C	0.27	0.67 (0.27, 1.64)	0.3791	0.43 (0.14, 1.29)	0.1303
rs9863627	PAK2	chr3:196808928	G	0.09	2.13 (0.66, 6.82)	0.2036	2.13 (0.66, 6.82)	0.2036
rs7720838	PTGER4	chr5:40486794	G	0.38	1.55 (0.71, 3.37)	0.2731	2.06 (0.64, 6.63)	0.2236
rs4848306	IL1B	chr2:112840530	A	0.45	0.77 (0.39, 1.51)	0.4443	2.00 (0.65, 6.14)	0.224
rs4469949	CD27	chr12:6436445	A	0.38	0.91 (0.45, 1.83)	0.7824	0.57 (0.23, 1.41)	0.2241
rs2071304	SPI1	chr11:47350826	G	0.25	1.18 (0.53, 2.61)	0.6909	1.89 (0.66, 5.39)	0.2357
rs152112	ITK	chr5:157217613	T	0.28	0.62 (0.26, 1.50)	0.2889	0.54 (0.18, 1.56)	0.2522
rs7574070	STAT4	chr2:191145762	A	0.32	0.67 (0.31, 1.48)	0.3219	0.58 (0.21, 1.55)	0.2738
rs17001247	CXCL10	chr4:75949138	C	0.26	1.72 (0.86, 3.42)	0.1238	1.63 (0.63, 4.23)	0.3186
rs10422141	TICAM1	chr19:4833398	T	0.49	0.58 (0.30, 1.11)	0.0999	0.64 (0.24, 1.65)	0.3533
rs4808137	UBA52	chr19:18578037	A	0.50	0.63 (0.33, 1.19)	0.1551	0.64 (0.23, 1.82)	0.4042
rs2276645	ZAP70	chr2:97713589	T	0.31	1.21 (0.64, 2.29)	0.5678	1.42 (0.56, 3.63)	0.4611
rs7584870	SOCS5	chr2:46746261	A	0.43	0.75 (0.39, 1.43)	0.3812	1.40 (0.50, 3.94)	0.5188
rs6673928	IL19	chr1:206763900	T	0.23	1.26 (0.58, 2.77)	0.5618	1.36 (0.51, 3.61)	0.5386
rs1149901	GATA3	chr10:8052824	T	0.21	0.71 (0.28, 1.78)	0.4646	0.75 (0.28, 2.04)	0.5788
rs6692729	PSEN2	chr1:226831218	T	0.44	0.88 (0.43, 1.83)	0.7376	0.76 (0.27, 2.10)	0.5965
rs665241	FYB	chr5:39266460	C	0.40	1.18 (0.63, 2.23)	0.6009	1.20 (0.47, 3.08)	0.7043

rs9895554	SKAP1	chr17:48046280	C	0.10	0.87 (0.29, 2.56)	0.7986	0.88 (0.29, 2.66)	0.8222
rs4402765	IL1A	chr2:112811270	C	0.27	0.81 (0.34, 1.96)	0.6424	0.91 (0.34, 2.41)	0.8435
rs11919943	CCR1	chr3:46225235	C	0.09	0.87 (0.25, 2.96)	0.82	0.89 (0.25, 3.11)	0.8506
rs8101605	LILRB1	chr19:54637036	A	0.21	1.29 (0.66, 2.54)	0.4595	1.08 (0.42, 2.83)	0.8702
rs2295359	IL23R	chr1:67170267	A	0.30	0.89 (0.38, 2.07)	0.7834	0.93 (0.32, 2.67)	0.8932
rs1551565	CAMK4	chr5:111225941	C	0.26	0.74 (0.32, 1.71)	0.4828	0.94 (0.36, 2.48)	0.9067
rs7036417	SYK	chr9:90808223	T	0.35	0.58 (0.30, 1.13)	0.1105	0.95 (0.37, 2.44)	0.9193
rs1378940	CSK	chr15:74791153	G	0.36	1.11 (0.53, 2.36)	0.778	1.05 (0.39, 2.86)	0.9241
rs10760142	C5	chr9:121073276	C	0.46	0.87 (0.49, 1.53)	0.624	0.96 (0.40, 2.33)	0.9323
rs4577037	IL16	chr15:81304319	G	0.05	0.97 (0.14, 6.54)	0.9769	0.97 (0.14, 6.54)	0.9769
rs11203203	UBASH3A	chr21:42416077	A	0.40	1.12 (0.58, 2.17)	0.7384	0.99 (0.36, 2.71)	0.9813
rs11161590	BCL10	chr1:85299315	G	0.41	1.03 (0.53, 2.00)	0.9245	1.00 (0.37, 2.71)	0.998
rs1049337	CAV1	chr7:116560533	T	0.26	0.77 (0.37, 1.61)	0.4932	1.00 (0.41, 2.43)	0.9981

* Models adjusted for age at pathological diagnosis, sex (male vs. female), Ashkenazi Jewish status (yes vs. no), primary tumor histologic subtype (superficial-spreading vs. nodular vs. desmoplastic vs. acral-lentiginous vs. lentigo-maligna vs. other), and AJCC staging at diagnosis

Supplemental Table 6: Variant associations for survival among SPMs, under the additive and dominant models*

SNP	Gene	SNP Position (GRCh38.p12)	Alternate Allele in our Population	Alternate allele frequency SPM patients	Hazard Ratio (95% C.I.)	*p*-value	Hazard Ratio (95% C.I.)	*p*-value
					Additive Model		*Dominant Model*	
rs2291299	CCL5	chr17:35864402	G	0.18	1.59 (1.15, 2.19)	**0.0048**	1.77 (1.18, 2.65)	**0.0057**
rs6673928	IL19	chr1:206763900	T	0.23	0.61 (0.43, 0.88)	0.0085	0.56 (0.37, 0.85)	0.0062
rs10422141	TICAM1	chr19:4833398	T	0.47	1.37 (1.04, 1.81)	0.0252	1.82 (1.13, 2.93)	0.0137
rs6695772	BATF3	chr1:212708597	C	0.39	1.18 (0.91, 1.54)	0.2173	1.65 (1.08, 2.50)	0.0197
rs11203203	UBASH3A	chr21:42416077	A	0.42	0.74 (0.56, 0.98)	0.0389	0.64 (0.43, 0.95)	0.0275
rs4577037	IL16	chr15:81304319	G	0.08	0.56 (0.31, 1.02)	0.0589	0.51 (0.26, 1.00)	0.0491
rs4796105	CCL5	chr17:35758948	C	0.15	1.39 (1.00, 1.93)	0.053	1.50 (1.00, 2.24)	0.0513
rs9895554	SKAP1	chr17:48046280	C	0.09	1.55 (0.97, 2.48)	0.0659	1.57 (0.95, 2.60)	0.0784
rs6692729	PSEN2	chr1:226831218	T	0.43	0.79 (0.58, 1.08)	0.1346	0.72 (0.48, 1.08)	0.1117
rs11569345	CD40	chr20:46129882	A	0.03	0.37 (0.09, 1.53)	0.1677	0.37 (0.09, 1.53)	0.1677
rs12401573	SEMA4A	chr1:156176427	C	0.42	1.24 (0.96, 1.60)	0.1008	1.33 (0.89, 1.99)	0.1691
rs4500045	PAG1	chr8:81105697	A	0.49	1.26 (0.96, 1.65)	0.0926	1.38 (0.86, 2.22)	0.1807
rs2701652	IRAK3	chr12:66187097	C	0.28	0.68 (0.51, 0.92)	0.0106	0.77 (0.52, 1.13)	0.1861
rs1551565	CAMK4	chr5:111225941	C	0.26	1.17 (0.87, 1.59)	0.3009	1.29 (0.88, 1.91)	0.1926
rs1049337	CAV1	chr7:116560533	T	0.28	1.04 (0.75, 1.43)	0.8309	1.25 (0.85, 1.84)	0.2526
rs152112	ITK	chr5:157217613	T	0.31	0.93 (0.67, 1.28)	0.6556	0.80 (0.53, 1.20)	0.2723
rs7584870	SOCS5	chr2:46746261	A	0.37	1.22 (0.92, 1.63)	0.1724	1.25 (0.83, 1.89)	0.2875
rs8101605	LILRB1	chr19:54637036	A	0.16	1.24 (0.86, 1.80)	0.2537	1.25 (0.82, 1.90)	0.2921
rs4469949	CD27	chr12:6436445	A	0.38	0.83 (0.62, 1.10)	0.1958	0.83 (0.56, 1.23)	0.3535
rs9921791	MLST8	chr16:2200067	T	0.08	0.79 (0.47, 1.34)	0.389	0.80 (0.47, 1.36)	0.4119
rs2276645	ZAP70	chr2:97713589	T	0.34	0.79 (0.58, 1.08)	0.1412	0.87 (0.58, 1.30)	0.4867
rs10760142	C5	chr9:121073276	C	0.45	1.07 (0.81, 1.41)	0.633	1.16 (0.77, 1.75)	0.4889
rs2295359	IL23R	chr1:67170267	A	0.32	1.05 (0.79, 1.40)	0.7187	1.15 (0.77, 1.72)	0.4961
rs9863627	PAK2	chr3:196808928	G	0.10	0.86 (0.55, 1.36)	0.523	0.88 (0.53, 1.44)	0.6019
rs1149901	GATA3	chr10:8052824	T	0.21	0.95 (0.67, 1.35)	0.7703	0.90 (0.59, 1.36)	0.6041
rs3807383	GIMAP5	chr7:150737070	C	0.25	0.95 (0.69, 1.31)	0.7526	0.90 (0.61, 1.34)	0.6147

rs7036417	SYK	chr9:90808223	T	0.34	1.09 (0.84, 1.42)	0.509	1.10 (0.74, 1.64)	0.6208
rs841718	STAT6	chr12:57099213	C	0.41	1.13 (0.83, 1.54)	0.4543	1.13 (0.70, 1.81)	0.6213
rs665241	FYB	chr5:39266460	C	0.47	0.98 (0.74, 1.30)	0.8839	0.89 (0.56, 1.42)	0.63
rs4808137	UBA52	chr19:18578037	A	0.47	0.88 (0.67, 1.16)	0.3801	1.12 (0.70, 1.79)	0.6479
rs7574070	STAT4	chr2:191145762	A	0.36	0.88 (0.65, 1.18)	0.3912	0.91 (0.61, 1.36)	0.6524
rs17001247	CXCL10	chr4:75949138	C	0.23	0.93 (0.67, 1.30)	0.6669	0.93 (0.62, 1.39)	0.7096
rs11919943	CCR1	chr3:46225235	C	0.10	0.89 (0.54, 1.48)	0.664	0.92 (0.54, 1.55)	0.7469
rs11161590	BCL10	chr1:85299315	G	0.45	1.19 (0.89, 1.58)	0.2381	0.94 (0.60, 1.45)	0.7689
rs859	IL16	chr15:81308981	G	0.28	1.07 (0.79, 1.45)	0.6599	1.05 (0.72, 1.55)	0.7941
rs4848306	IL1B	chr2:112840530	A	0.41	0.93 (0.71, 1.21)	0.5969	0.95 (0.64, 1.42)	0.8038
rs1378940	CSK	chr15:74791153	G	0.39	1.00 (0.75, 1.33)	0.9884	1.03 (0.69, 1.54)	0.878
rs7720838	PTGER4	chr5:40486794	G	0.42	1.19 (0.89, 1.60)	0.2351	1.03 (0.68, 1.57)	0.8893
rs2071304	SPI1	chr11:47350826	G	0.35	0.97 (0.73, 1.30)	0.8538	1.02 (0.68, 1.53)	0.9103
rs13331952	CKLF	chr16:66549715	C	0.12	0.93 (0.59, 1.46)	0.7569	0.98 (0.59, 1.62)	0.9323
rs4402765	IL1A	chr2:112811270	C	0.27	0.95 (0.68, 1.32)	0.7435	0.99 (0.67, 1.45)	0.941

* Models adjusted for age at pathological diagnosis, sex (male vs. female), Ashkenazi Jewish status (yes vs. no), primary tumor histologic subtype (superficial-spreading vs. nodular vs. desmoplastic vs. acral-lentiginous vs. lentigo-maligna vs. other), and AJCC staging at diagnosis

Supplemental Figure 1: Principal component analysis (PCA) identification of cancer-free controls with AJ ancestry

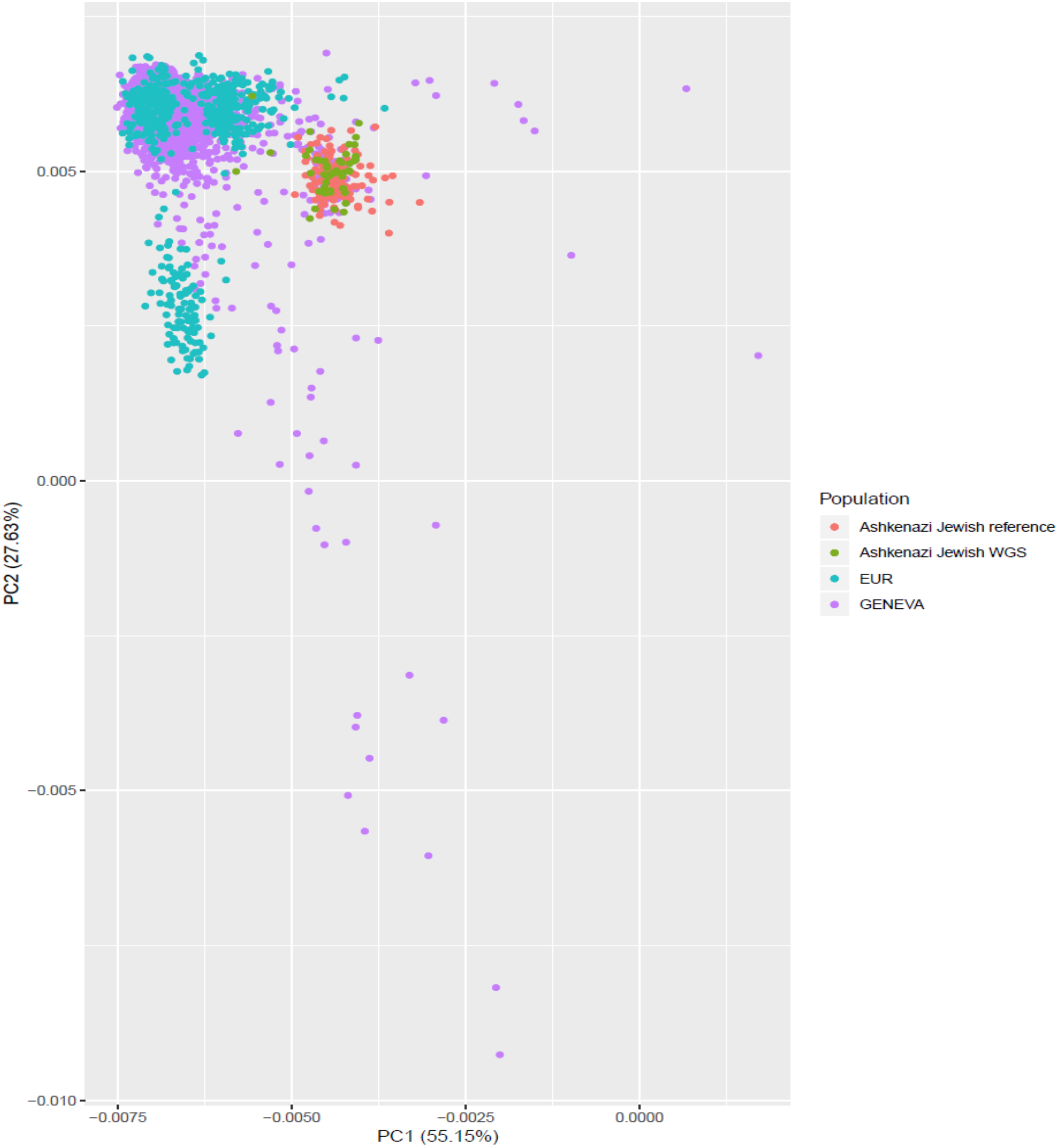

Supplementary Figure 1: Principal component analysis on 34,507 SNPs in linkage equilibrium demonstrating population stratification between patients with known Ashkenazi Jewish reference population (red), Ashkenazi Jewish patients in our study with WGS information (green) and European (blue) ancestry. Patients from the GWAS (phs000187.v1.p1) control population (purple) were identified as putatively of AJ ancestry if they had PC1 in interval [-0.005, -0.003754] and PC2 in interval [0.00375, 0.00625].
